# The Frequency of Subarachnoid Hemorrhage from Very Small Cerebral Aneurysms (< 5 mm): A Population-Based Study

**DOI:** 10.7759/cureus.279

**Published:** 2015-06-18

**Authors:** Parviz Dolati, Daniel Pittman, William F Morrish, John Wong, Garnette R. Sutherland

**Affiliations:** 1 Neurosurgery, Beth Israel Deaconess Medical Center; 2 clinical neurosciences, University of Calgary; 3 Department of Neurosurgery, University of Calgary

**Keywords:** small aneurysm, rupture, frequency, rate, subarachnoid hemorrhage

## Abstract

Background: The prevailing view amongst neurosurgeons is that the larger the aneurysm, the higher the chance of rupture. This implies that very small aneurysms rarely rupture. To investigate this theory, we conducted a cross-sectional hospital-based study of aneurysmal subarachnoid hemorrhage, with an emphasis on aneurysm size at the time of rupture.

Methods: We retrospectively reviewed hospital records and radiological tests of all patients admitted to Foothills Medical Center, Calgary, Alberta, with a ruptured saccular aneurysm from January 2008 to January 2012. The size of the dome and neck (in millimeters), the aspect ratio (aneurysm depth to aneurysm neck), and location of the aneurysms were determined using preoperative computed tomography angiography and digital subtraction angiography.

Findings: One hundred and twenty-three patients with a ruptured saccular aneurysm were identified. The average size of the dome, neck, and the aspect ratio was 6.6±4.4 mm (range: 1.5-26 mm), 3.1 mm, and 2.6±0.9, respectively. Forty-six patients (37%) had a ruptured aneurysm with dome size < 5 mm (range: 1.5-4.9 mm). For these small aneurysms, the average size of the dome, neck, and the aspect ratio was 3.9+1.1 mm, 1.6 mm, and 2.1+0.6, respectively. The anterior communicating artery was the most common location regardless of size.

Conclusion: Small aneurysms (< 5 mm) are a common cause of aneurysmal subarachnoid hemorrhage. When unruptured, looking for other risk factors for rupture is highly recommended before simply leaving them alone.

## Introduction

Controversy exists in the management of patients with unruptured intracranial aneurysms less than 5 mm in diameter. The prevalence of intracranial aneurysms in the general population is about 3% with reports varying from 0.2% to 9.9% [1]. On the other hand, the incidence of aneurysmal subarachnoid hemorrhage (SAH) is much lower at 6 to 10 cases per 100,000 per year [1-5]. The 30-day mortality rate of aneurysmal SAH is as high as 45% with 50% of the survivors sustaining irreversible brain injury [4]. These poor outcomes have produced arguments in favor of intervention prior to aneurysm rupture.

The first phase of a large multicentric international study of unruptured intracranial aneurysms, the International Study of Unruptured Intracranial Aneurysms (ISUIA-1, 1998), revealed surprising results [1]. Among patients with no previous history of SAH and aneurysms < 10 mm in diameter, the risk of rupture was less than 0.05% per year [1]. In those with a past history of SAH, the rate was ten times higher at 0.055% per year [1]. For aneurysms > 10 mm, the risk of rupture was approximately 1% per year.

The second phase of the ISUIA was also surprising [6]. The five-year cumulative rupture rate among patients without a prior history of SAH, and with an aneurysm < 7 mm, was 0% for anterior circulation aneurysms and 2.5% for aneurysms within the posterior circulation. This study showed that the larger the size of the aneurysm, the higher the cumulative rupture rate. The authors concluded that asymptomatic aneurysms less than 7 mm in diameter in the anterior circulation were benign.

Most recently, the Unruptured Cerebral Aneurysm Study (UCAS) in a Japanese cohort, which gathered prospective data on the natural history of 5,720 patients with 6,697 aneurysms, yielded results similar to the ISUIA [7]. In the UCAS, larger aneurysm size was associated with a higher hazard ratio for the rupture. However, this study had a significant selection bias. More than 2,000 of its unruptured small aneurysms had been treated surgically. Therefore, aneurysms with possibly a high potential for rupture may have been treated prior to rupture. Moreover, not all eligible cases were enrolled in the study.

However, several investigators have contradicted these studies, reporting a higher percentage of small aneurysms among their case series of ruptured intracranial aneurysms [3, 5, 8-12]. This indicates a discrepancy between the ISUIA and UCAS data and the size of ruptured aneurysms seen in routine clinical practice.

In order to better understand the significance of small aneurysms and subarachnoid hemorrhage, this study reviewed patients presenting with subarachnoid hemorrhage to the Foothills Medical Center. The Foothills Medical Center receives all patients with subarachnoid hemorrhage throughout Southern Alberta.

## Materials and methods

The Institutional Review Board of the University of Calgary approved this study (protocol #25227). The hospital records, database registry, and radiological tests of all patients who were admitted at the Foothills Medical Center with a ruptured saccular aneurysm from January 2008 to January 2012 were included in this retrospective review. Other types of aneurysms, including mycotic or traumatic, were excluded. The size of the dome and neck (in millimeters) and the location of the aneurysms were determined using the standard measurement device on preoperative computed tomography angiography (CTA) and digital subtraction angiography (DSA). According to the maximum diameter of the dome of the ruptured intracranial aneurysms (RIAs) and our observation of many RIAs < 5 mm, patients were subdivided into two major groups: Group A with small RIAs < 5 mm and Group B with RIAs with a maximum dome diameter > 5 mm. Patients were treated with direct aneurysmal clipping, coiling, or conservatively.

For statistical evaluations, the size of the ruptured aneurysms was used as the dependent variable. Several determinant factors (true smoking, hypertension, blood pressure, alcohol or drug intake, age, sex, location, and clinical presentation) were evaluated using a Chi-squared test to assess their association with aneurysm size (α=0.05).

## Results

The size of aneurysms in relation to the clinical and demographic characteristics is shown in Table 1. Forty-three (34%) were men and 80 (66%) were women with an overall mean age of 56.6 years (range: 21-79 years). There was a significant difference in mean age according to sex (male: 52.3, SD=12.5, female: 59, SD=14, p-value, 0.001). There was no difference in the distribution of Group A and B ruptured aneurysms in different age groups (p=0.577).

**Table 1:** Size of ruptured aneurysms in relation to clinical and demographic characteristics

	Group A	Group B
	Aneurysms < 5 mm (N=46)	Aneurysms > 5 mm (N=77)
Mean age (SD)	55.2 (10)	55.4 (11)
Sex		
Male	12 (9.7%)	31 (25%)
Female	34 (28%)	46 (37.3%)
Risk Factors		
Smoking	10 (23%)	28 (36%)
Hypertension	7 (17%)	23 (30%)
Smoking and hypertension	0	11 (14%)
Past history of SAH	3 (6%)	2 (2.5%)
Heavy alcohol drinker	0	1 (1.2%)
Strong family history of SAH	0	3 (3.9%)
History of cocaine abuse	1 (2%)	0
No known risk factors	28 (60%)	35 (45%)
Location of Ruptured Aneurysm		
AComA	23 (18.7%)	22 (17.8%)
MCA bifurcation	10 (8%)	16 (13%)
PComA	2 (1.6%)	9 (7.3%)
Basilar tip	1 (0.8%)	8 (6.5%)
Other anterior circulation	7 (5.6%)	17 (14%)
Other posterior circulation	3 (2.4%)	5 (4%)
Location of Ruptured Aneurysm		
Anterior circulation	43 (35%)	64 (52%)
Posterior circulation	3 (2.4%)	13 (10.6%)
Multiplicity of Aneurysm in One Patient		
Single aneurysm	43 (35%)	72 (58.6%)
Multiple aneurysms	3 (2.4%)	5 (4%)
Hunt and Hess Grades of Clinical Presentation		
Grade 1	16 (35%)	26 (34%)
Grade 2	6 (17%)	24 (31%)
Grade 3	5 (11%)	15 (20%)
Grade 4	5 (11%)	8 (10%)
Grade 5	12 (26%)	4 (5%)

The frequency of the associated risk factors with the RIAs is presented in Table 2. About 60% of RIAs < 5 mm and 45% of RIAs > 5 mm were not associated with any known risk factor (p=0.136). A Chi-square test between RIA size and the co-morbidities of smoking and hypertension individually showed no statistically significant association in the two groups (p=0.447 and p=0.051, respectively). However, the combination of these two comorbidities, smoking plus hypertension, was shown to significantly differentiate between the two groups (p=0.007).

**Table 2:** Association of potential risk factors with ruptured aneurysm in two groups

Risk Factor	In RA<5 m (N/%)	In RA>5 mm	P-Value
Cigarette smoking	10 (23%)	28 (36%)	0.447
Poorly controlled hypertension	7 (17%)	23 (30%)	0.051
Smoking and hypertension	0 (0%)	11 (14%)	0.004
No known risk factor	28 (60%)	35 (45%)	0.136
Clinical presentation with Hunt and Hess Grade 4 and 5	17 (37%)	12 (15%)	0.011

Sixty-three percent of patients in Group A and 85% of patients in Group B presented with a Hunt and Hess Grade 1-3 SAH. Patients with RIAs < 5 mm were associated with a higher rate of presentation with a poorer Hunt and Hess Grade (4 and 5) than those with RIAs > 5 mm (p=0.011).

Thirty-eight patients out of these 123 were treated by surgical clipping, 80 via endovascular coiling, and five died as a result of their initial hemorrhage prior to surgical or endovascular intervention. The overall mean size of the dome, neck, and AR was 6.6 mm (SD=4.4, range: 1.5-26 mm), 3.1 (SD=2), and 2.7 (SD=0.9), respectively. Forty-six patients (37%) had RIAs with the maximum dome size < 5 mm. For these small aneurysms, the average size of the dome, neck, and AR were 3.8 mm (SD=1.1, range: 1.5-4.9 mm), 3.1 mm (SD=2), and 1.6 (SD=0.8), respectively. The frequency of RIAs with different sizes is shown in Table 3. Ninety-eight (80%) of ruptured aneurysms had a maximum dome diameter of < 10 mm. Ruptured aneurysms in the anterior communicating artery (AComA) were significantly smaller than those in the basilar tip or ICA termination.

**Table 3:** Frequency of ruptured intracranial aneurysms based on their location and the size of ruptured aneurysms.

Aneurysm Location	Aneurysm < 5 mm	Aneurysm > 5-10 mm	Aneurysm > 10 mm	Total	Mean Size of Rupture
AComA	23 (50%)	19 (36.5%)	3 (12%)	45 (36.6%)	5+2.5 mm
MCA bifurcation	10 (21.7%)	9 (17.3%)	7 (28%)	26 (21.1%)	7.4+4.7 mm
PComA	2 (4.3%)	9 (17.3%)	0 (0%)	11 (8.9%)	5.9+1.1 mm
Basilar tip	1 (2.2)%	0 (0%)	8 (32%)	9 (7.3%)	13.9+5.9 mm
Other anterior circulation	7 (15.2%)	10 (19.3%)	7 (28%)	24 (62.5%)	7.2+5.1 mm
Other posterior circulation	3 (6.6%)	5 (9.6%)	0 (0%)	8 (13.2%)	4.4+1.8 mm
Total	46 (37%)	52 (42%)	25 (21%)	123 (100%)	6.6+4.4 mm

**Figure 1:**
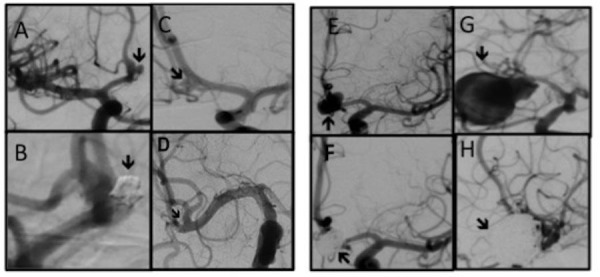
Digital subtraction angiography (DSA) of different sized ruptured aneurysms. A and B (both AP views), pre- and post-coiling of a 2 mm anterior communicating artery (AComA) aneurysm. C (AP view) and D (Submentovertex view), pre- and post-clipping of a 2.5 mm right middle cerebral artery (MCA) aneurysm. In Fig. D, the arrow points to the clip’s tip. E and F (both AP views), Pre- and post-coiling of a 9 mm AComA aneurysm, and finally, G and H (both oblique views), pre- and post-coiling of a 26 mm right MCA aneurysms. They had presented with SAH Grade 4, 3, 2, and 1, respectively.

Table 3 shows the frequency of ruptured intracranial aneurysms based on their location and size of the rupture. Ninety-four percent of aneurysms < 5 mm and 83% of aneurysms > 5 mm were located in the anterior circulation. In the posterior circulation, the overall frequency of aneurysms was very low, although most of them had a diameter > 5 mm. The anterior communicating artery was the most common location of the RIAs irrespective of the size, followed by the middle cerebral artery. Some examples of small versus larger ruptured aneurysms are presented in Figure 1.

## Discussion

Subarachnoid hemorrhage from a RIA is associated with high morbidity and mortality [1, 4, 6, 13-14]. However, due to the reported significant risks associated with the prophylactic treatment of unruptured intracranial aneurysms (UIA), the optimal management of small (< 5 mm) aneurysms remains controversial. In the ISUIA, the perioperative morbidity and mortality for patients who underwent either clipping or coiling of their unruptured intracranial aneurysm was about 10% [9, 14].

Many have criticized the surprising result of the ISUIA-1 due to the calculation of rupture rate by analysis of their retrospective component (Group 1) [1]. The ISUIA-2 was also controversial, as its reported rupture rate was much lower than previous and subsequent reports [8, 11-12, 15-18]. The frequency of small aneurysms and the average size of rupture of previously reported clinical and autopsy series are shown in Table 4.

**Table 4:** The frequency of small aneurysms and the average size of rupture of previously reported clinical and autopsy series.

Author/Year	Number of RIAs		Size of Ruptured Intracranial Aneurysm				Average Rupture Size
			< 5 mm	5-10 mm		> 10 mm	
1- Molyneux [4] 2002		Coiled: 1073	552 (51%)	438 (41%)		83 (8%)	N/A
		Clipped: 1070	572 (53%)	426 (40%)		72 (7%)	N/A
2- Weibers et al. [6] 2003		Group 1: 41 (No history of SAH)	2 < 7 mm	5: 7.9 mm		34 > 9 mm	10.9 mm
		Group 2: 8 (With history of SAH)	7 < 7 mm	1: 7.9 mm			N/A
3-Morita et al. [7], 2012		111	N/A, but the higher the size, the higher the rupture rate and hazard ratio.				N/A
4 - Forget, et al. [8] 2001		245	86(35%)		124 (50.6%)	35 (14.4%)	N/A
5 - Joo, et al. [9] 2008		627	451 (71.8%)		552 (88%) < 10 mm	6.3 mm	6.3 mm
6- Ohashi, et al. [11] 2004		280	73 (26.1%)		135 (48.2%)	72 (25.7%)	7.6 mm
7 - Orz, et al. [12] 1997		1248	475 (38%) (< 6mm)		681 (54.5%) (6-15mm)	82 (6.5%) >15mm	6.7mm
8- Graf et al. [13] 1974		1092	142 (13%)		775 (71%) < 10 mm	175 (16%)	8.2 mm
9- Beck, et al. [17] 2006		83	50 (60%)		68 (82%) < 10 mm	6.7 mm	6.7 mm
10 - Rosenørn, et al. [18] 1993		908	162 (18%)		474 (52%)	272 (30%)	N/A
11 - Weir, et al. [19] 2002		812	50 (6%)		495 (61%)	268 (33%)	10.8 mm
12 - Inagawa, et al. [21] 1990		109	18 (17%)		50 (46%)	41 (38%)	9.5 mm
13 - MC Cormic, et al. [25] 1970		191	159 (83%) < 10 mm		32 (17%)		N/A
14- Yasui, et al. [27] 1996		25	16 (64%)		6 (24%)	3 (12%)	N/A

Kassell, et al. [10, 16] had found that 13% of their RIAs were < 5 mm and 57% were between 5 and 10 mm. Based on these findings, they concluded that UIAs < 10 mm should not be considered safe, and UIAs > 5 mm should be repaired surgically. Orz, et al. [12], Forget, et al. [8], and Weir, et al. [19] reported significantly different from others. In these studies, the proportion of the RIAs < 10 mm was 93%, 85·6%, and 67%, respectively. Moreover, one year earlier, the International Subarachnoid Aneurysm Trial (ISAT) [4] showed that 51% out of 1,073 of their RIAs that were treated by coiling and 53% out of 1,070 of RIAs that underwent clipping, were < 5 mm. Overall, in ISAT, more than 90% of RIAs in either group were < 10 mm.

Since the publication of the ISUIA study, several other authors have reported small aneurysms as a significant proportion of their RIAs [1, 10, 17]. Among these studies, the report of Joo, et al. [9] was notably different from the ISUIA results. They reported 627 RIAs, of which 451 (71.8%) of cases were < 5 mm, and that overall, 552 (88%) RIAs were < 10 mm. This report, along with other similar reports, reinforced the existing controversy.

The proportion of the small aneurysms in our study was even higher than many previous reports, including Kassell, et al. [10], Rosenørn, et al. [18], Forget, et al. [8], Ohashi, et al. [11], and the International Cooperative Study [13]. The mean sizes of rupture herein were 5+2.5 mm and 7.4+ 4.7 mm for AComA and middle cerebral artery (MCA) aneurysms, respectively. The overall mean size in our series was 6.6+4.4 mm. The mean size of the previously reported RIA was 8-10 mm, 13-49% of which were smaller than 5-6 mm [5, 8, 11-12, 20]. Ohashi, et al. [11] reported 280 RIA, with 208 aneurysms (74.3 %) < 10 mm and 26.1 % < 5 mm. The mean size of the RIAs in their report was 7.6 mm.

Care must be taken when applying post-mortem observations to the clinical setting. In an autopsy  study of 133 post-mortem cases, Inagawa, et al. [21] reported 109 RIAs, of which 18 (17%) were < 4 mm in diameter, 50 (46%) were 5-9 mm in diameter, and 41 (37%) were > 10 mm in diameter. Based on this data, Inagawa concluded that larger aneurysms are more likely to rupture and also were associated with more extensive hemorrhage. However, the findings of our current clinical study differ from those of the autopsy studies. The current study shows that patients with RIAs < 5 mm are associated with a higher rate of presentation with poor Hunt and Hess grades than RIAs > 5 mm (p=0.011). Moreover, in the clinical setting, as shown in the present study, approximately half of the RIAs < 5 mm and 65% of RIAs > 5 mm are clinically presented with a good Hunt and Hess SAH grade. In other words, most patients survive from even severe SAH.

Some authors speculate that the size of the dome of an aneurysm will shrink after the rupture. However, there is no definitive angiographic and histological evidence supporting this argument. In a histological study of ruptured intracranial aneurysms [21] and another study, which compared the size of the RIAs before and after the rupture [22-23], no shrinkage of the ruptured aneurysm was reported. Forget, et al. [8] have presented angiographic and histopathological evidence that aneurysms do not shrink after rupture. Furthermore, Beck, et al. [17] observed no cases of a decrease in the size of aneurysm after intra-angiographic rupture of their cases.

As we demonstrated in this series, ruptured aneurysms in the AComA were significantly smaller than those in the basilar tip or internal carotid artery (ICA) termination. This indicates that AComA aneurysms may rupture with a much smaller size or in the earlier stages of their development. These results differ from the ISUIA study, yet are in agreement with many other studies, such as those reported by Beck, et al. [17], Weir, et al. [19], Ohashi, et al. [11], and Forget, et al. [8], in which ruptured aneurysms were much more common in the anterior circulation, and especially in the AComA. Our results, similar to the UCAS, are that the posterior circulation aneurysms were not more prone to rupture than anterior circulation aneurysms, and that the aneurysms most prone to rupture were more often located in AComA and PComA [7]. UCAS also showed aneurysms in both of these locations have a relatively high rate of rupture even with a size of < 7 mm, whereas ISUIA reported a minimal rupture rate in patients with no history of SAH and anterior circulation aneurysm < 7 mm [14, 18].

Association between the risk factors and a RIA is another area of controversy within the literature. In our series, about 60% of RIAs < 5 mm and 45% of those > 5 mm were not associated with any known risk factor. Similarly, Ohashi, et al. [11] found no significant difference in RIAs size between smokers and nonsmokers (7.5 mm and 7.8 mm in smokers and nonsmokers, respectively). On the other hand, the proportion of RIAs < 5 mm was significantly higher amongst patients with poorly controlled hypertension as compared to normotensives. This diverges from the Cooperative Study of Intracranial Aneurysms [13] in which the ruptured aneurysms were reported to be larger in hypertensive patients. Some other studies, however, found no association between hypertension and the size of ruptured aneurysms [24-27]. Overall, there is no consistency in the reports, which discuss these correlations.

In the current study, patients with a RIAs < 5 mm had a greater association with poorer Hunt and Hess Grades (4 and 5) than those with RIAs > 5 mm (p=0.011). It has long been recognized that small aneurysms found when unruptured behave differently from those presenting with SAH. There are various possible explanations for this. One is that small aneurysms are high-risk when they first form and either bleed quickly and present as SAH or stabilize to be found as unruptured low-risk aneurysms later. Another is that there are really two different phenotypes of small aneurysms, one unsafe that presents with SAH and one safe that turn up incidentally.

## Conclusions

Small aneurysms (< 5 mm) are a common cause of aneurysmal subarachnoid hemorrhage. When unruptured, looking for other risk factors for rupture is highly recommended before simply leaving them alone.
